# American Nervousness—Its Philosophy and Treatment

**Published:** 1879-08

**Authors:** George M. Beard

**Affiliations:** New York.


### ARTICLE IV.

### *American Nervousness: Its Philosophy and Treatment.*

BY GEORGE M. BEARD, M. D., NEW YORK:

**An Address delivered before the Baltimore Medical and Surgical Society, February 12th, 1879.**

Gentlemen:—If our fathers in medicine of the last century could be brought from their graves to this room this evening, and be told of the subject that has brought us together, the first question they would ask would be, "What is meant by the term nervousness?" They would say, and very truly, that the Greeks had no word for nervousness as we now understand that term; and that even down to the eighteenth century, nervousness was supposed to mean irritability of temper, disposition to anger, excitability—a mental quality, and not a physical disease. In reply, we should be obliged to say that in the nineteenth century, nervousness meant nervelessness, nervous exhaustion, abnormal susceptibility of the nervous system to internal or external irritants.

American nervousness, during the past half century, has expressed itself by a large variety of symptoms, a number of which are so frequent, so positive in their character, and so important, that they have given names to disease, and are known as such. Among these symptoms and expressions of modern nervousness are neuralgia, sick headache, nervous dyspepsia, hay fever, and, above all, *neurasthenea*, or nervous exhaustion in all its various forms. These conditions, with others that might be mentioned, constitute a family of nervous diseases that have developed chiefly during the last half century—at least, during the present nineteenth century—and are most abundant, and most severe and most varied in their manifestations in the northern portion of the United States, although they are found in, and are now extending to England and the Continent of Europe.

The rise of this family of functional nervous diseases brings a new era into medicine and sociology, for it has no precedent in the history of mankind. The ancients had no nervous disease, or almost none, save a few cases of insanity and epilepsy here and there; and our moderns knew little or nothing about them until the present century.

The scientific proofs of this unprecedented nervousness of the Americans during this generation are very numerous. I will mention but a few.

First of all, there is the *increased sensitiveness to cold and*

*heat*, which is observed among all our brain-working classes. Our fathers were content with a temperature of 60° F. We must have, to be comfortable, a temperature of at least 70° ; and there are many families who keep their rooms at even a much higher temperature. In other words, we are 10 degrees more sensitive to cold than were our fathers. The heat of our summers is no greater than it was a century ago ; but the cases of sunstroke and heat prostration are widely out of proportion to the increase in our population.

Note also our sensitiveness to stimulants and narcotics, as alcohol and tobacco, and even tea and coffee. Not only our fathers but our mothers, could drink freely of wines and stronger liquors, and even smoke as much as they wished, without developing any of the nervousness of our time. At the present time, a very considerable proportion of the population of this country are unable to smoke, or chew, or drink even mild wine, or tea or coffee—especially the latter—without making themselves perceptibly worse thereby. I find that a very considerable number of my nervous patients have been compelled, before I see them, to give up their coffee and tobacco. All this is modern and pre-eminently American. Likewise the idiosyncrasies of patients in regard to the action of medicines and the effects of drugs and various external irritants, have during the last half century, multiplied in variety and phase, and greatly augmented in number. There are thousands who cannot bear opium—who are kept awake instead of being put to sleep by it. The ordinary dose for an adult is sufficient to deprive them of a night's repose. One very eminent physician finds that even chocolate, one of the mildest beverages, is a poison to him ; and another experienced physician who consulted me one time in regard to himself, could not, he said, bear anything that I prescribed. I spoke of iron ; he said iron, even in small doses made his head ache ; and when I tried it, even with other medicines, it produced that effect. I suggested quinine ; he said quinine made him crazy. I tried a zinc combination ; it disturbed his stomach. And yet this man, so variously sensitive, was actively engaged in one of our most laborious professions.

One of the very best signs of our civilization is found in the *premature decay of our teeth.* Special explanations, without number, have been offered for this long-observed phenomenon—such as the use of sweets, the use of acids, neglect of cleanliness, and the use of food that requires little mastication. But they who urge these special facts to account for the decay of teeth of our civilization would, by proper inquiry, learn that the savages and negroes, and semi-barbarians everywhere, in many cases use sweets far more than we, and never clean their mouths, and never suffer, except in old age, from cavities in the teeth. The cause of the decay of teeth is subjective far more than objective—in the constitution of the modern civilized man. Similarly, also, with regard to irregularities of the teeth, which, as is now known, ar· dependent on bad nutrition of the jaws.

Delicacy of digestion is one of the best known and first observed effects of civilization upon the nervous system. The history of the *rise and fall of pork as a food* is itself most instructive on this point. Pork, like the Indian, flees before civilization. In all the great cities of the East, among the brain-working classes of our large cities everywhere, pork in all its varieties and preparations has taken a subordinate place among the meats upon our tables, for the reason that the stomach of the brain-worker cannot digest it. Three times a day, and every day in the year almost, pork in some form was the only dependence of our fathers in the last generation, who could eat it freely without ever asking themselves whether it was easy or hard to be digested. This dethronement of pork has had, and is still having a disastrous effect upon the American people; for, as yet, no article of food with a sufficient amount of fat has been generally substituted; and fat in our dietaries is one of the most imperative hygienic needs in our time, and which has become to be felt, both instinctively and rationally, and which, on all hands, we are trying to meet by the use of cream, cod-liver oil, eggs, fish, and the fats of fresh meat.

The *eyes*, also, are good barometers of our nervous civilization. The increase of asthenopia and shortsightedness, and, in general, of the functional disorders of the eye, are demonstrated facts and are most instructive. The great skill and great number of our oculists are constant proof and suggestions of the nervousness of our age.

In this sensitiveness of organization, the *reproductive system* ever shares. One of the many evils of our time, we are told, is, that the habit of self-abuse is on the increase, and that men are more indulgent than formerly. Hence the increase of nervous diseases that are connected with the genital functions; and hence the terrific results that sometimes follow early begun and long-continued masturbation. But so far as can be learned from all sources of information on these difficult themes, it would appear that among savages and the semi-civilized, sexual abuse, both in a natural and unnatural way, is carried to a higher degree, on the average, than among the civilized; we cannot, indeed, bear these abuses as our fathers could. The observation of Bulwer, that it requires a strong constitution to be dissipated, is a just and sound one. The modern young man is not strong enough to abuse himself as perhaps he would be willing to do, or as his ancestor did. Both natural and unnatural methods of sexual indulgence react with fearful and almost immediate power on the nervous system, with symptoms which, through the labors of charlatans, have become familiar as the songs of infancy. In marital intercourse, we are compelled, as a rule, to exercise a caution and a moderation of which our ancestors knew nothing.

The world inquires why diseases of women increase, and many special causes are assigned; but the one great cause, to which all others are subordinate, is civilization.

The question often agitated is, Whether diseases have changed their type in modern times? This is a question which should not be discussed; to raise it, is to answer it. There is no question but that diseases have changed their type in the last half century. The only question is, What are the degrees of the change, and what are the causes which produce these results?

It is demonstrable that nervous diseases have increased in recent periods; and that, with this increase of nervous symptoms, there has been also an increase in the asthenic forms of disease, and a decrease in the sthenic forms; and, correspondingly, that there has been a change in the methods of treatment of diseases; that neurasthenia—nervous susceptibility—has affected all, or nearly all, diseases, so that nearly all illnesses occurring among the better class of people—the brain-workers—require a different kind of treatment from that which our fathers employed for the same diseases.

The four ways by which we determine these facts are—*first*, by studying the literature of medicine of the past centuries; *secondly*, by conversation with very old and experienced practitioners—men between the ages of seventy and ninety—who link the past with the present generation, and remember their own personal experience and the practice of medicine as it was fifty years ago; *thirdly*, from our own individual experience and observation; *fourthly*, by studying the habits and diseases of savages and barbarians of all climes and ages, and of the lower orders about us.

Statistics on this subject are of very little value, for reasons that will be clear to those who are used to statistics, and who know how they can be handled. Longevity has increased almost *pari passu* with this increase of nervousness and change in type of disease, and this has been a stone of stumbling and rock of offence to those who have discussed this subject. Both facts are true; longevity has increased among the brain-working classes, and nervousness has also increased. These two apparently opposite facts are harmonized by a third factor which those who have studied this subject failed to reach—namely, nervousness is not only consistent with longevity, but actually favors it, by preserving the system from attacks of acute inflammatory disease. We do not bear blood-letting now as our fathers did for the same reasons that we do not bear alcohol, tobacco, coffee, opium, physical pain and sexual indulgence as they could. The change in the treatment of disease is a necessary result of the change in the modern constitution. The old-fashioned constitution yet survives in numbers of people; and in such cases, the old treatment is oftentimes better than the modern treatment.

The diseases of savages can be learned from books of travel and from conversations with travelers. Many of these books, it is true, are of an non-expert character, so far as the diseases of the savage tribes are concerned; but some of them are written by physicians and scientific men of various degrees of eminence, whose observations on a large scale, compared together, enable us to arrive at the approximate truth. In the study of this subject, I have compared a very large number of books of travel, and I have arrived at this fact, in regard to which there can be no doubt whatever, namely, that nervous disease scarcely exists among savages or barbarians, or semi-barbarians, or partially civilized people. Likewise, in the lower orders in our great cities, and among the peasantry in the rural districts, muscle-workers, as distinguished from brain-workers—those who represent the habits and mode of life and diseases of our ancestors of the last century—nervous diseases, except those of an inflammatory or syphilitic character, are about as rare as they were among all classes during the last century. These people frequenty need more violent and severe purging, more blood-letting, more frequent blistering than the higher orders would endure. If we would compare the nervous diseases of our time with those of the past, we have only to look about us among those classes of people whose temperaments take us back a half or three-quarters of a century; in these classes such diseases as neurasthenia, heavy fever, sick headache are very rare indeed; so that it is very difficult for a hospital for nervous diseases to succeed in getting a sufficient number of patients of this character. On the other hand, hospitals for inflammatory and febrile diseases are enormously patronized among them. It is partly for this reason that the literature for nervous functional diseases is so poor and unsatisfactory. Our medical books and lectures are made up far too often of hospital, charity and dispensary practice.

[TO BE CONTINUED.]

